# Recurrence quantification analysis of resting state EEG signals in autism spectrum disorder – a systematic methodological exploration of technical and demographic confounders in the search for biomarkers

**DOI:** 10.1186/s12916-018-1086-7

**Published:** 2018-07-02

**Authors:** T. Heunis, C. Aldrich, J. M. Peters, S. S. Jeste, M. Sahin, C. Scheffer, P. J. de Vries

**Affiliations:** 1 Department of Mechanical and Mechatronic Engineering, Stellenbosch University, Stellenbosch, South Africa; 2 Division of Child and Adolescent Psychiatry, University of Cape Town, 46 Sawkins Road, Rondebosch, 7700 South Africa; 3 Department of Mining Engineering and Metallurgical Engineering, Western Australian School of Mines, Curtin University, Perth, Australia; 4 Department of Process Engineering, Stellenbosch University, Stellenbosch, South Africa; 5 Division of Epilepsy and Clinical Neurophysiology, Department of Neurology, Boston Children’s Hospital, Boston, USA; 6 Semel Institute of Neuroscience and Human Behavior, David Geffen School of Medicine, University of California Los Angeles, California, USA; 7 Translational Neuroscience Center, Department of Neurology, Boston Children’s Hospital and Harvard Medical School, Boston, USA

**Keywords:** Autism spectrum disorder, Resting state electroencephalography, Recurrence quantification analysis, RQA

## Abstract

**Background:**

Autism spectrum disorder (ASD) is a neurodevelopmental disorder with a worldwide prevalence of 1–2%. In low-resource environments, in particular, early identification and diagnosis is a significant challenge. Therefore, there is a great demand for ‘language-free, culturally fair’ low-cost screening tools for ASD that do not require highly trained professionals. Electroencephalography (EEG) has seen growing interest as an investigational tool for biomarker development in ASD and neurodevelopmental disorders. One of the key challenges is the identification of appropriate multivariate, next-generation analytical methodologies that can characterise the complex, nonlinear dynamics of neural networks in the brain, mindful of technical and demographic confounders that may influence biomarker findings. The aim of this study was to evaluate the robustness of recurrence quantification analysis (RQA) as a potential biomarker for ASD using a systematic methodological exploration of a range of potential technical and demographic confounders.

**Methods:**

RQA feature extraction was performed on continuous 5-second segments of resting state EEG (rsEEG) data and linear and nonlinear classifiers were tested. Data analysis progressed from a full sample of 16 ASD and 46 typically developing (TD) individuals (age 0–18 years, 4802 EEG segments), to a subsample of 16 ASD and 19 TD children (age 0–6 years, 1874 segments), to an age-matched sample of 7 ASD and 7 TD children (age 2–6 years, 666 segments) to prevent sample bias and to avoid misinterpretation of the classification results attributable to technical and demographic confounders. A clinical scenario of diagnosing an unseen subject was simulated using a leave-one-subject-out classification approach.

**Results:**

In the age-matched sample, leave-one-subject-out classification with a nonlinear support vector machine classifier showed 92.9% accuracy, 100% sensitivity and 85.7% specificity in differentiating ASD from TD. Age, sex, intellectual ability and the number of training and test segments per group were identified as possible demographic and technical confounders. Consistent repeatability, i.e. the correct identification of all segments per subject, was found to be a challenge.

**Conclusions:**

RQA of rsEEG was an accurate classifier of ASD in an age-matched sample, suggesting the potential of this approach for global screening in ASD. However, this study also showed experimentally how a range of technical challenges and demographic confounders can skew results, and highlights the importance of probing for these in future studies. We recommend validation of this methodology in a large and well-matched sample of infants and children, preferably in a low- and middle-income setting.

## Background

There has been growing interest in the development and evaluation of biomarkers in a range of health-related conditions to aid in the identification of individuals at risk, to support diagnosis, to identify subgroups and to monitor treatment [1, 2]. There is consensus that an ideal biomarker should be easily accessible, affordable, accurate, and have high sensitivity and specificity for the particular health condition under investigation [1–3]. In global healthcare, a biomarker that can screen and identify those at risk for a condition for which early intervention is available could be particularly valuable. In such a scenario, the biomarker should be able to classify an individual as ‘at risk’ in comparison to population-based peers.

Autism spectrum disorder (ASD) has a global prevalence estimate of 1–2% in children [4–8]; 90% of people with ASD live in low- and middle-income countries [9], where there is a significant demand for low-cost screening tools that do not require highly trained professionals [3, 9–11]. Early identification is an essential first step to prevent unnecessary delays in access to early intervention strategies, parent education and planning for longer-term support [12–15].

There has been positive progress regarding development, validation and implementation of screening questionnaires, such as the Modified Checklist for Autism in Toddlers (M-CHAT), as early screening tools for ASD [16–18]. However, the cultural appropriateness of rating scale measures, the language and literacy demands, and the fact that rating scales only identify difficulties when development or behaviour already have noticeable, albeit subtle, changes, makes global implementation of such tools problematic. The development of ‘language free, culturally fair’ screening tools that may use contemporary technology therefore holds great promise for global screening in ASD [10, 19].

Given the interest in early identification, there has been growing interest in biomarkers for ASD. In a thought-provoking perspective piece, Walsh et al. [1] warned against overenthusiasm in the attempts to identify ASD biomarkers. They warned that (1) the manifestations of ASD unfold over time, and therefore a biomarker that is age- and developmentally sensitive would be challenging to identify; (2) many proposed biomarkers have poor sensitivity, namely they are not good at classifying those with ASD as ASD, and also have poor specificity, wherein the biomarker is also associated with other neurodevelopmental disorders; and (3) many of the current proposed biomarkers are expensive, complicated and reliant on high levels of technical expertise, thus limiting their potential implementation in clinical settings.

There has been growing interest in electroencephalography (EEG) as an investigational tool for biomarker development in neurodevelopmental disorders. In a recent scoping review, we highlighted two distinct but related areas that pose challenges for EEG as potential biomarkers for ASD and related neurodevelopmental disorders [3]. We described a range of potential demographic, clinical and technical confounders including age, sex, intellectual ability, socioeconomic status, comorbidity, the use of medication, eyes-open versus eyes-closed condition, the number and location of electrodes, and test-retest reliability, all of which will require evaluation before EEG biomarkers can be deemed sufficiently robust for translation into a clinical setting for assessment of risk at an individual level. Secondly, we described key technical challenges in the identification of appropriate multivariate, next-generation analytical methodologies that can characterise the complex, nonlinear dynamics of neural networks in the brain [2, 20–22]. Three novel potential biomarkers for ASD risk have been proposed by Bosl et al. [22], Duffy and Als [13], and Pistorius et al. [23]. The review by Heunis et al. [3] provides a detailed methodological comparison of these three novel rsEEG biomarkers.

In short, Bosl et al. [22] applied modified multiscale entropy (MME) analysis to rsEEG data to compare infants at high risk for ASD (HRA; defined as having an older sibling with ASD) and typically developing (TD) infants. Accuracies of 80–100% were achieved with subsamples ranging from 12 to 28 subjects in total, aged 6–9 months. Accuracy declined from 12 to 24 months of age, likely reflecting that subjects in the high risk group went on to develop more typical brain function. The findings by Bosl et al. [22] were criticised after publication, primarily given the knowledge that only a modest proportion of infants at HRA will go on to develop ASD. Critics therefore argued that, until the HRA group had been confirmed to have or not to have ASD at, for instance, the age of 3, no firm conclusions about the MME rsEEG biomarker should be drawn [24].

Duffy and Als [13] proposed spectral coherence analysis (CA) as a biomarker for ASD. The sample population investigated comprised 430 children with a confirmed diagnosis of ASD and 554 TD children aged 2–12 years. Analysis revealed an average classification success of 86% for the ASD group and 88.5% for the TD group. Improved performance was noted for the more restricted age subsamples, potentially reflecting age-related changes in rsEEG. The CA biomarker method appeared to be useful in the binary categorical classification of ASD versus typical development, but many clinical and analytical questions regarding biomarker development remained unanswered in this regard.

We previously proposed recurrence quantification analysis (RQA) of rsEEG as a novel biomarker of ASD risk [3, 23]. RQA is an emerging nonlinear data analysis technique in the field of EEG applications. The technique is based on the fundamental property of recurrence inherent to complex systems such as the brain [25]. RQA of EEG signals has been successfully applied to discriminate between sleep stages and to characterise different behaviours of sleep EEG recordings in patients with sleep apnea syndrome [26], to assess the depth of anaesthesia [27] and in the automated identification of epileptic EEG signals [21]. In our proof-of-principle study for ASD [23], we investigated RQA in a sample of 7 ASD and 5 TD subjects, aged 8–17 years, with analysis of 12 best segments. Results showed 83.3% accuracy, 85.7% sensitivity and 80% specificity with a linear discriminant analysis (LDA) classifier [23]. A further study, by a different group [28], used RQA in search of a biomarker for ASD using 12 10-s segments of a single channel of task-related EEG from a 20-year-old participant with ASD and a 29-year-old TD individual.

The aim of this study was to conduct a replication and extension of the proof-of-principle study in a larger sample, and to investigate the robustness of the potential RQA biomarker in the context of a systematic methodological exploration of a number of technical and demographic variables that may act as covariates or confounders. We set out to refine biomarker parameters, to evaluate the biomarker against potential confounders such as age, sex and intellectual ability, to determine the accuracy, sensitivity and specificity, and to explore test-retest reliability of the RQA biomarker. A clinical scenario of diagnosing an unseen subject was simulated using a leave-one-subject-out classification approach. The novelty of this work therefore lies in the multivariate application of RQA to rsEEG for the early detection of ASD risk, and in the systematic evaluation of potential technical and demographic confounders on accuracy, sensitivity and specificity.

## Methods

### Recurrence analysis

A recurrence plot (RP) enables the visualisation of higher dimensional phase spaces in a two-dimensional plot. The visual appearance of a RP is used to characterise the underlying dynamics of a system. A recurrence event is calculated, according to Eq. 1, for each sample combination *i* and *j* of time series *x* and a specified threshold distance *ε* (neighbourhood size), and is stored in an *N* x *N* matrix used to construct a RP [29]. In a RP at coordinates (*i*, *j*), black dots are plotted when recurrence events (*R*_*i*, *j*_ ≡ 1) occur, and white dots in the case of nonevents (*R*_*i*, *j*_ ≡ 0).

1 $$ {R}_{i,j}=\left\{\begin{array}{c}1:\kern1.75em \left\Vert {x}_i-{x}_j\right\Vert \le \varepsilon \\ {}0:\kern1.75em otherwise\kern2.25em \end{array}\right.\kern1.75em i,j=1,\dots, N $$

The application of RQA to RPs provides an objective means of quantifying system dynamics. Several geometric features can be extracted from RPs, such as recurrence rate (RR; the probability that any state will recur), determinism (DET; indicative of the predictability of the system), entropy (ENTR; providing a measure of complexity of the recurrence structure) and laminarity (LAM; the probability that a state will not change for the next time step). Marwan et al. [29] and Schinkel et al. [25] provide further mathematical detail for RPs and RQA features.

### Subjects

De-identified rsEEG data were obtained from Boston Children’s Hospital, Harvard Medical School, Boston, and the Semel Institute, University of California, Los Angeles, USA [30]. The relevant ethics approval was obtained to allow secondary analysis of this data; primary data analysis was published by Peters et al. [30]. The dataset comprised rsEEG from 16 non-syndromal ASD participants, aged 2–6 years, and 46 TD participants, aged 0–18 years. Age, sex and level of intellectual ability (categorised as none, mild, severe or unknown) was known for each subject. ASD diagnoses were made by board-certified paediatric neurologists using the Diagnostic and Statistical Manual of Mental Disorders (DSM-IV-TR), and in most cases included an Autism Diagnostic Observation Schedule (ADOS) performed by clinical or research-reliable administrators [30]. All children in their study were assessed by full developmental and neuropsychological assessment. For study purposes, all clinical and neuropsychological data were combined by the lead paediatric neurologist to make a categorical classification of Intellectual Disability. TD subjects were selected from the general neurology clinic at Boston Children’s Hospital. Subjects who had been referred for a single clinical event of moderate-to-low suspicion for epilepsy (for instance, syncope, tics, behavioural outbursts, headache and prominent startle), but who showed normal neurological development for age, had a normal physical examination, had a normal EEG during wakefulness and sleep, and had a clinical follow-up of at least 1 month to confirm the trivial nature of the EEG referral, were included as control subjects [30].

The demographic characteristics and sample composition of the populations studied are summarised in Table 1. It was possible to match subjects on a pair-wise basis for age, but not for sex and intellectual ability.

**Table 1:** Demographic characteristics and sample composition of populations studied

Description	Subjects		Number of segments	Age range (years)		Mean age (years)		Sex ratio (male:female)	
	ASD	TD		ASD	TD	ASD	TD	ASD	TD
Full sample	16	46	4802	2–6	0–18	4.06	7.94	3:1	1:1.4
Subsample	16	19	1874	2–6	0–6	4.06	1.98	3:1	1:1.4
Age matched	7	7	666	2–6	2–6	3.96	3.93	2.5:1	1:1.3

*ASD* Autism spectrum disorder, *TD* typically developing

### EEG signal processing methodology

**Fig. 1:**
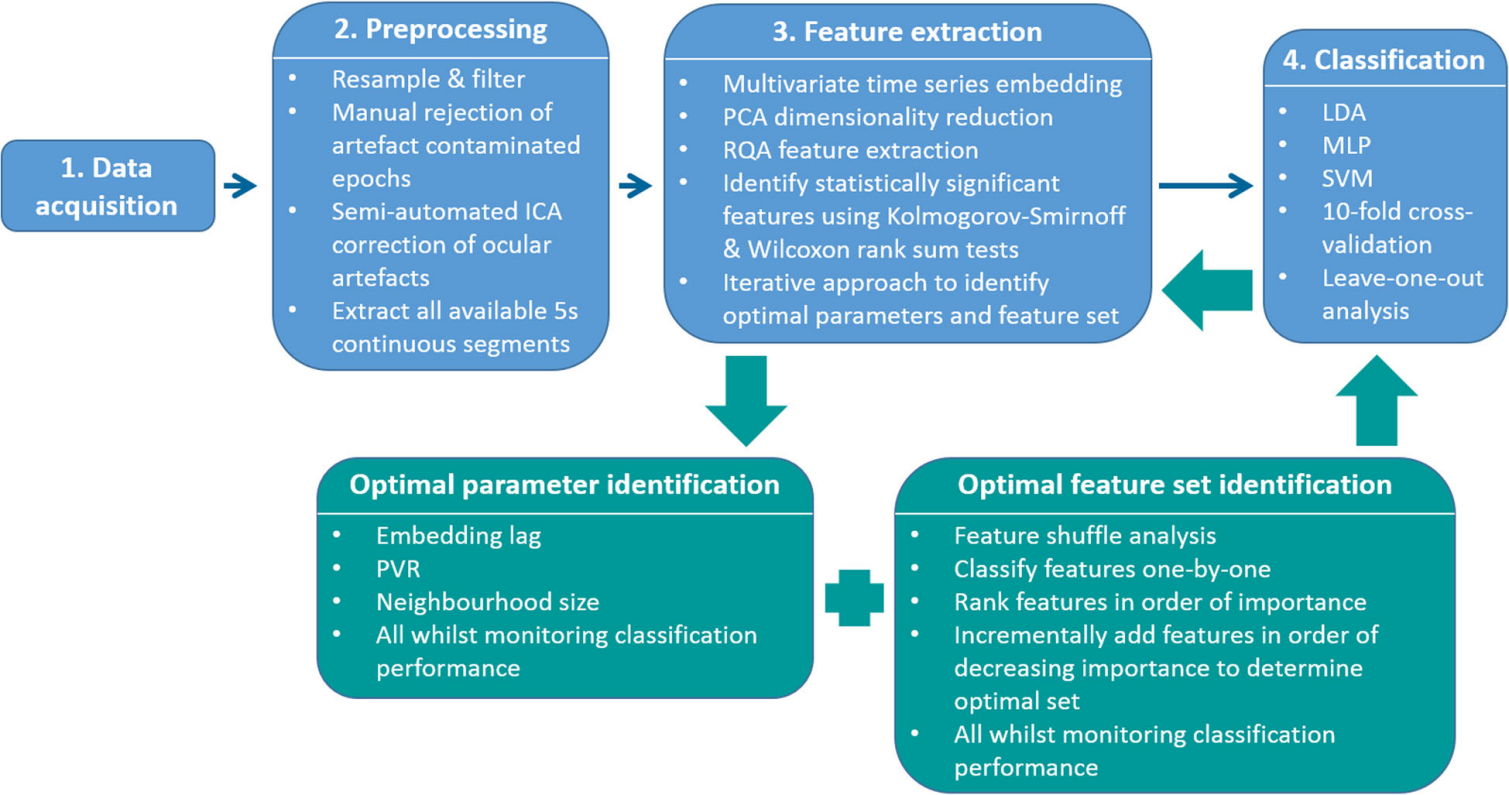
EEG signal processing methodology

The EEG signal processing steps implemented are discussed according to a signal processing pipeline of data acquisition, preprocessing, feature extraction and classification (Fig. 1). The review paper by Heunis et al. [3] provides a general description of the steps in a typical EEG signal processing pipeline.

### Data acquisition

The full dataset comprised 16 ASD (mean age 4.06 years, 3:1 male to female ratio) and 46 TD subjects (mean age 7.94 years, 1:1.4 male to female ratio). EEG records were collected retrospectively from either routine clinical EEG recordings or long-term EEG monitoring with video. Data from Boston Children’s Hospital were recorded using either a Biologic recording system (256–512 Hz sampling rate, 0.1–100 Hz band pass range) or a Natus Neuroworks system (200 Hz sampling rate, 0.1–100 Hz band pass range), using 19 standard electrodes for a clinical setting (Fp2, Fp1, F4, F3, Fz, C4, C3, Cz, P4, P3, Pz, F8, F7, T8, T7, P8, P7, O2, O1). Data from University of California, Los Angeles were collected using a 128 Hydrocel Geodesic Sensor Net System (EGI, Inc.) and NetAmps Amplifiers and NetStation software (250 Hz sampling rate), and digitised with a National Instruments Board (12 bit). All EEG systems used the 10–20 system for electrode placement.

### Data pre-processing

Long segments of EEG data were clipped from the raw EEG, and epochs containing many artefacts were removed. The data were then notch filtered at 60 Hz, resampled to 200 Hz and spatially down-sampled to the standard clinical 19 electrodes (Fp2, Fp1, F4, F3, Fz, C4, C3, Cz, P4, P3, Pz, F8, F7, T8, T7, P8, P7, O2, O1), where relevant. Note that channels Fp1and Fp2 were excluded from further analysis as they primarily contain ocular artefact information; a total of 17 EEG channels were thus used in this multivariate analysis. An average reference was created using the BESA Research 3.5 software package. EEGLAB [31] was used to filter the data, using an FIR filter (1–70 Hz). A paediatric neurologist and board certified clinical neurophysiologist (JMP) inspected the data, manually rejected artefact-ridden epochs, and selected awake task-free data, of a minimum of 2 min in length [30]. Muscle-contaminated epochs were rejected where possible. Ocular artefact correction, to remove eye blinks and lateral eye movements, was performed using independent component analysis and EEGLAB [30]. The next step was to extract all available continuous 5-s segments per subject.

### Feature extraction

Multivariate embedding was used to construct a phase space representation of the EEG dynamics using the 17 EEG channels and Taken’s time delay embedding method [32]. A multi-channel lagged trajectory matrix was created per segment per subject, each column was embedded separately with the same lag and dimension and then all columns were horizontally concatenated to form the multi-channel lagged trajectory matrix. All available continuous 5-s segments per subject were used. Dimensionality reduction of each multi-channel lagged trajectory matrix was performed using principal component analysis (PCA) [33]. Each principal component score vector was used to reconstruct the attractor in a multidimensional phase space. The Cross Recurrence Plot MATLAB toolbox, developed by Marwan et al. [29], was used to plot RPs and to extract 10 RQA features from each of the multiple dimensionality reduced embedded segment matrices per subject. The 10 RQA features extracted were, as described above, RR, DET, mean diagonal line length, longest diagonal line, ENTR, LAM, trapping time, longest vertical line, recurrence time of the 1st Poincaré recurrence (T1) and recurrence time of the 2nd Poincaré recurrence (T2). A description of each feature is summarised in Table 1 of the proof-of-principle study by Pistorius et al. [23]. Statistical significance of features was determined using the Kolmogorov–Smirnoff and Wilcoxon rank sum tests (for distribution and shape) on the training data features.

An iterative approach was used to identify the optimal parameter and feature set combination. The combination that yielded the best classification performance was identified as optimal. Embedding lag, embedding dimension, percentage variance to retain (PVR) after PCA dimensionality reduction and RQA neighbourhood size were evaluated. The data from cross-validation run 1 and the ‘significant RQA feature set’ were used to determine the optimal parameter values; these values were used for all cross-validation runs.

Embedding lags of 15–25 were estimated using the first minimum of the average mutual information index, evaluated per channel. The corresponding optimal embedding dimension for each channel was computed using the false nearest neighbour method, using the Quick-Ident MATLAB toolbox [34], and yielded a value of 10. The choice of embedding parameters is a crucial decision in reconstructing an attractor in a phase space. However, with classification problems, or applications where one attempts to distinguish different groups, the parameter choice is less important, as long as the data are handled consistently. In testing the sensitivity of the PVR parameter, a range of 10–100 PVR with varying increments was evaluated. The neighbourhood investigated for the detection of recurrence events was defined using the maximum norm neighbourhood shape, the heuristic of ‘a few percent of the maximum phase space diameter’ [25] was used to determine the neighbourhood size. Neighbourhood sizes ranging from 2.0 to 4.0 in intervals of 0.1 were evaluated. The initial estimate of the neighbourhood size was 3.0, taking into account the abovementioned heuristic, findings from the Pistorius et al. [23] study and visual inspection of the recurrence plots.

After identification of the optimal parameter values, the optimal feature set was determined by performing feature shuffle analysis in order to confirm that all features were contributing useful discriminatory information to the classifiers for class membership prediction. This entailed shuffling the test labels of each feature one-by-one, and also all features at once, classifying the full feature set including the relevant feature(s) with shuffled labels, and then comparing the classification performance achieved with the shuffled feature sets to that of the unshuffled feature set. Further, each feature was classified one-by-one and ranked in order of feature importance. The optimal feature set was then determined by adding one feature at a time to the set in order of decreasing importance. The features required to achieve the best classification performance were identified as the optimal set.

For the purpose of this study, we chose to only include the statistically significant RQA features. However, this decision is questionable, as a feature may not be statistically significant between groups, but in combination with other features it may allow for clearer group distinction after classification. In an attempt to address some of the clinical challenges identified by Heunis et al. [3], we were able to investigate age and sex as covariates, given the limitations of the available data. The various feature sets tested were all significant RQA features (‘RQA’), combinatorial feature sets including the significant RQA features and demographic features (‘RQA + age’, ‘RQA + sex’, ‘RQA + age + sex’) and demographic features without RQA features (‘age’, ‘sex’ and ‘age + sex’). With the 10-fold cross-validation analyses, two feature set options were investigated – feature set 1 comprising all statistically significant RQA features, and feature set 2 comprising all statistically significant RQA and demographic features (including age and sex).

### Classification

Two classification approaches were implemented, namely 10-fold cross-validation and a leave-one-out (or leave-one-subject-out) approach. The cross-validation approach was used to optimise the parameter values, after which the leave-one-out approach was used to validate the cross-validation classification results and to simulate a clinical scenario and evaluate the outcome of ‘diagnosing’ a new/unseen subject. For the cross-validation approach, a total of 10 training and 10 test datasets were created. Each training and test dataset comprised a random selection of 70% training data and 30% test data from each subject. For the leave-one-out approach, considering the age-matched sample with 14 subjects (Table 1), 14 training and 14 test datasets were created. Each training dataset comprised 13 subjects, with a different test subject’s data being assigned to the test dataset with each of the 14 leave-one-out runs. Training and test data were standardised (mean of zero and standard deviation of one) as required. Three classification algorithms were implemented using MATLAB, namely LDA, a multilayer perceptron (MLP) neural network with nine nodes in one hidden layer, using the scaled conjugate gradient backpropagation training algorithm, and a support vector machine (SVM) with a nonlinear radial basis function kernel. Accuracy, sensitivity, specificity, sample size, number of segments and sample composition (the proportion of segments within each group) within the training and test data are reported in order to allow meaningful interpretation of the classification performance results.

### Sample population analysis

**Fig. 2:**
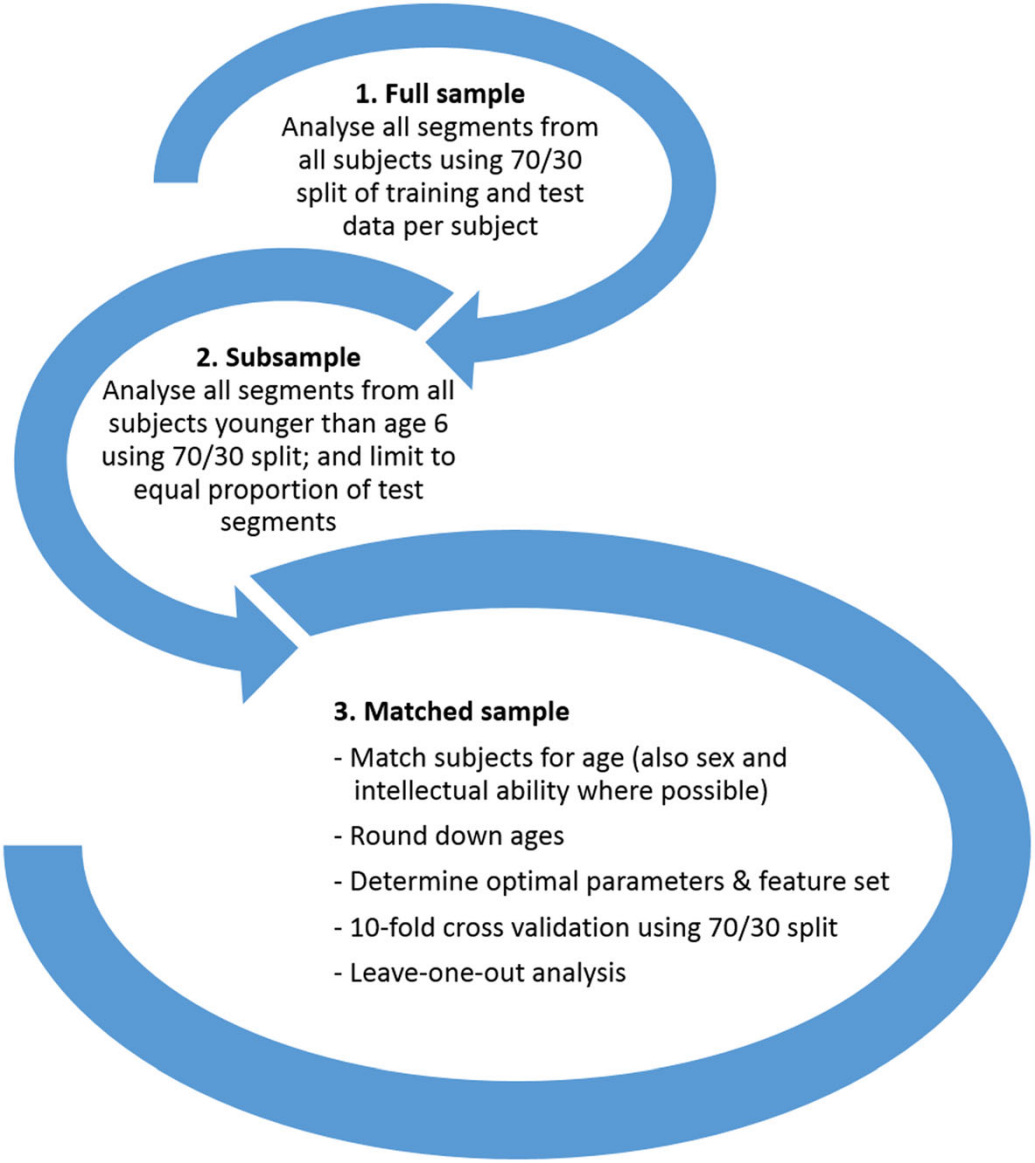
Progress loop of sample population analysis

A full sample, subsample and age-matched sample were evaluated (Fig. 2). The reasons for conducting the analyses in this way are discussed in the results section.

## Results

### Full sample: cross-validation approach

**Fig. 3:**
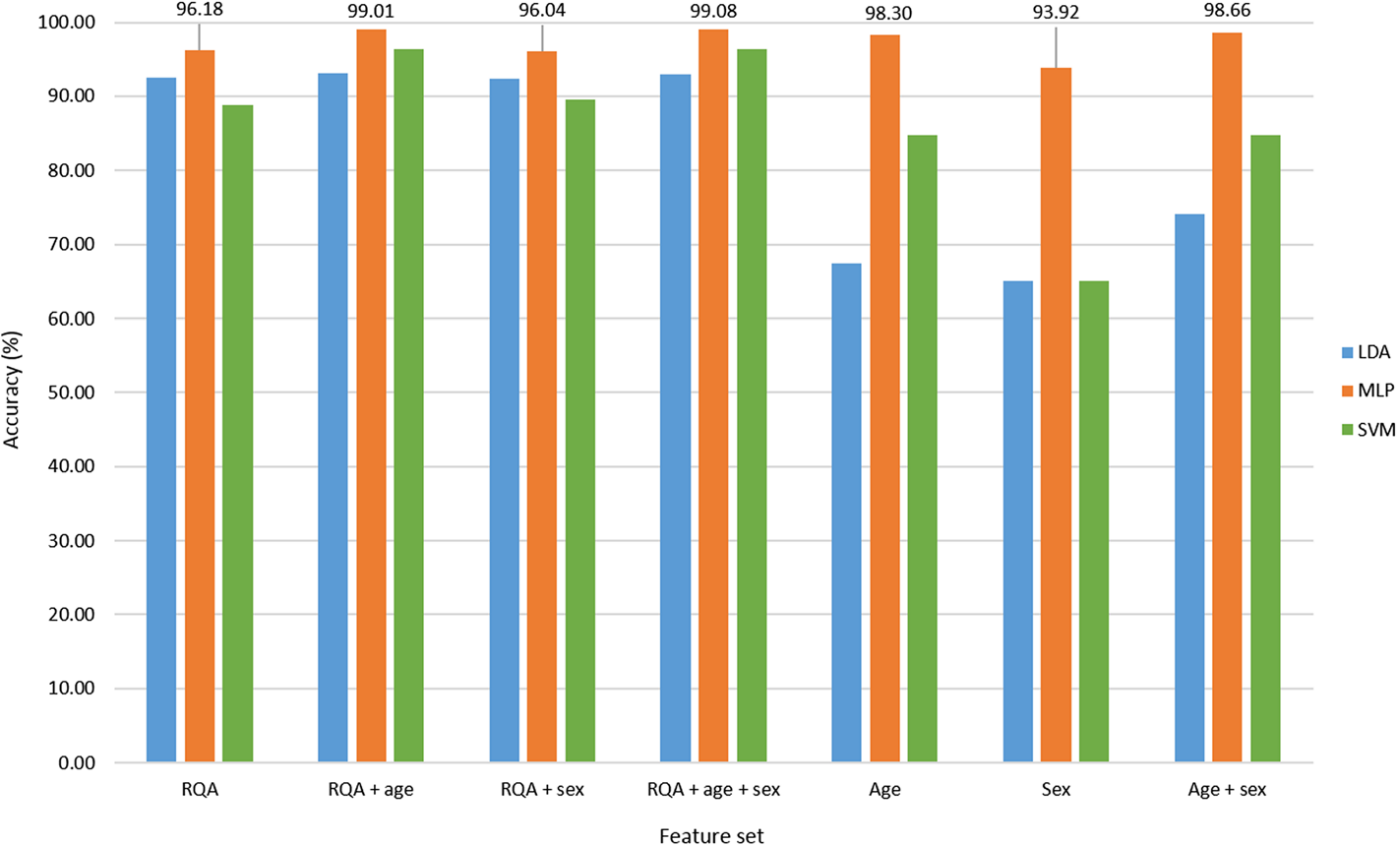
Classification accuracy of different feature sets for full sample cross-validation run 1

The optimal parameter set identified was comprised of an embedding lag of 25, embedding dimension of 10, PVR of 22.12 (equivalent to six principal components (PCs)) and a neighbourhood size of 3.0. Figure 3 provides an illustration of the classification accuracy achieved with the different feature sets for each of the classifiers investigated. The MLP classification results showed 96.18% accuracy with the ‘RQA’ feature set, and 99.08% accuracy with the combinatorial feature set ‘RQA + age + sex’. It seemed that age and sex were useful covariates in combination with the RQA feature set, contributing to increased classification accuracy. However, classification of the demographic features alone and in combination revealed 98.3% accuracy with ‘age’, 93.92% with ‘sex’ and 98.66% with ‘age + sex’. Given that age and sex in a random sample would not predict ASD or TD group membership, these spurious results were suggestive of a sample bias. To address this sample artefact, a subsample of all subjects younger than age 6 years was selected for further analysis. A further observation made in the full sample was that 93.9% of the test data rsEEG segments were TD and 6.1% ASD. To avoid misinterpretation of the classification results, in the subsample, an equal number of test segments per group were used for analysis such that there was a 50/50 chance for the classifier to guess group membership correctly.

### Subsample: cross-validation approach

**Fig. 4:**
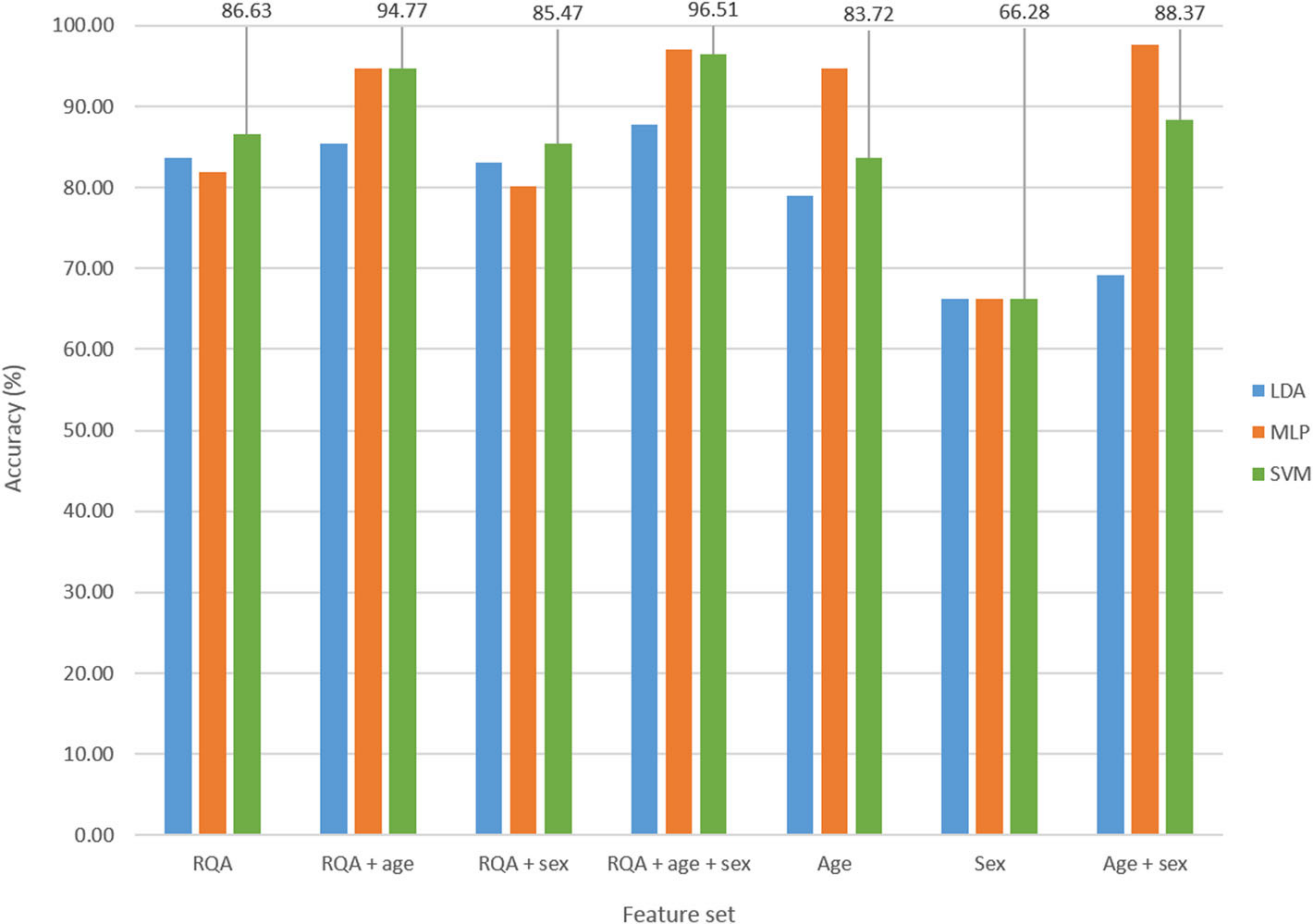
Classification accuracy of different feature sets for subsample cross-validation run 1

The optimal parameter set identified was comprised of an embedding lag of 25, embedding dimension of 10, PVR of 12.60 (equivalent to three PCs) and a neighbourhood size of 3.0. Figure 4 illustrates the classification accuracy achieved for the different feature sets and classifiers evaluated. The SVM classifier results are reported here, given that the SVM classifier achieved the highest accuracy of 86.63% on the ‘RQA’ feature set alone; 96.51% was achieved with the ‘RQA + age + sex’ feature set, 83.72% with ‘age’, 66.28% with ‘sex’, and 88.37% with ‘age + sex’. Given the distribution of age and sex within the subsample, the demographic features were still sufficient to classify ASD and TD subjects with higher accuracy than that achieved with the ‘RQA’ feature set. Sample bias therefore remained a problem. To address this, the next step was to analyse a matched sample. In addition, ages were rounded down to prevent the classifiers from predicting group membership based on exact age values encountered in the training data, e.g. 5.25 years and 5.41 years were both rounded down to 5 years.

### Age-matched sample: cross-validation approach

An embedding lag of 25, embedding dimension of 10, PVR of 30.09 (equivalent to 10 PCs) and a neighbourhood size of 2.9 were identified as optimal. This neighbourhood size amounted to approximately 6.7% of the average maximum phase space size (of 43.13) achieved for all cross-validation runs.

A summary of the mean and standard deviation values, along with the *p* values of the Kolmogorov–Smirnoff and Wilcoxon rank sum statistical tests conducted for the optimal feature set for cross-validation run 1 is provided in Table 2. It is important to keep in mind that, with each cross-validation run, a different random 70/30 split of training and test data was made, and possibly a different statistically significant feature set was identified. The clinical implication is that, once a robust range has been identified for each group within an ASD and TD sample, new/unseen subjects can be ‘diagnosed’ as ‘at risk for ASD’ or ‘TD’ depending on the range of values in which the newly extracted RQA features fall.

**Table 2:** Summary of feature set 1 for age-matched sample cross-validation run 1

Feature	ASD (mean ± standard deviation)	TD (mean ± standard deviation)	Kolmogorov–Smirnoff test (*p* value)	Wilcoxon rank sum test (*p* value)
RR	0.683 ± 0.0134	0.6756 ± 0.0109	< 0.0001	< 0.0001
DET	0.98 ± 0.0052	0.9858 ± 0.0043	< 0.0001	< 0.0001
ENTR	3.0956 ± 0.1553	3.1785 ± 0.1544	< 0.0001	0.0001
LAM	0.9861 ± 0.0038	0.9911 ± 0.0031	< 0.0001	< 0.0001
T1	1.4548 ± 0.0285	1.4725 ± 0.0245	< 0.0001	< 0.0001
T2	17.78 ± 2.738	19.57 ± 3.4224	< 0.0001	< 0.0001

*ASD* Autism spectrum disorder, *DET* determinism, *ENTR* entropy, *LAM* laminarity, *RR* recurrence rate, *T1* recurrence time of the first Poincaré recurrence, *T2* recurrence time of the second Poincaré recurrence, *TD* typically developing

**Fig. 5:**
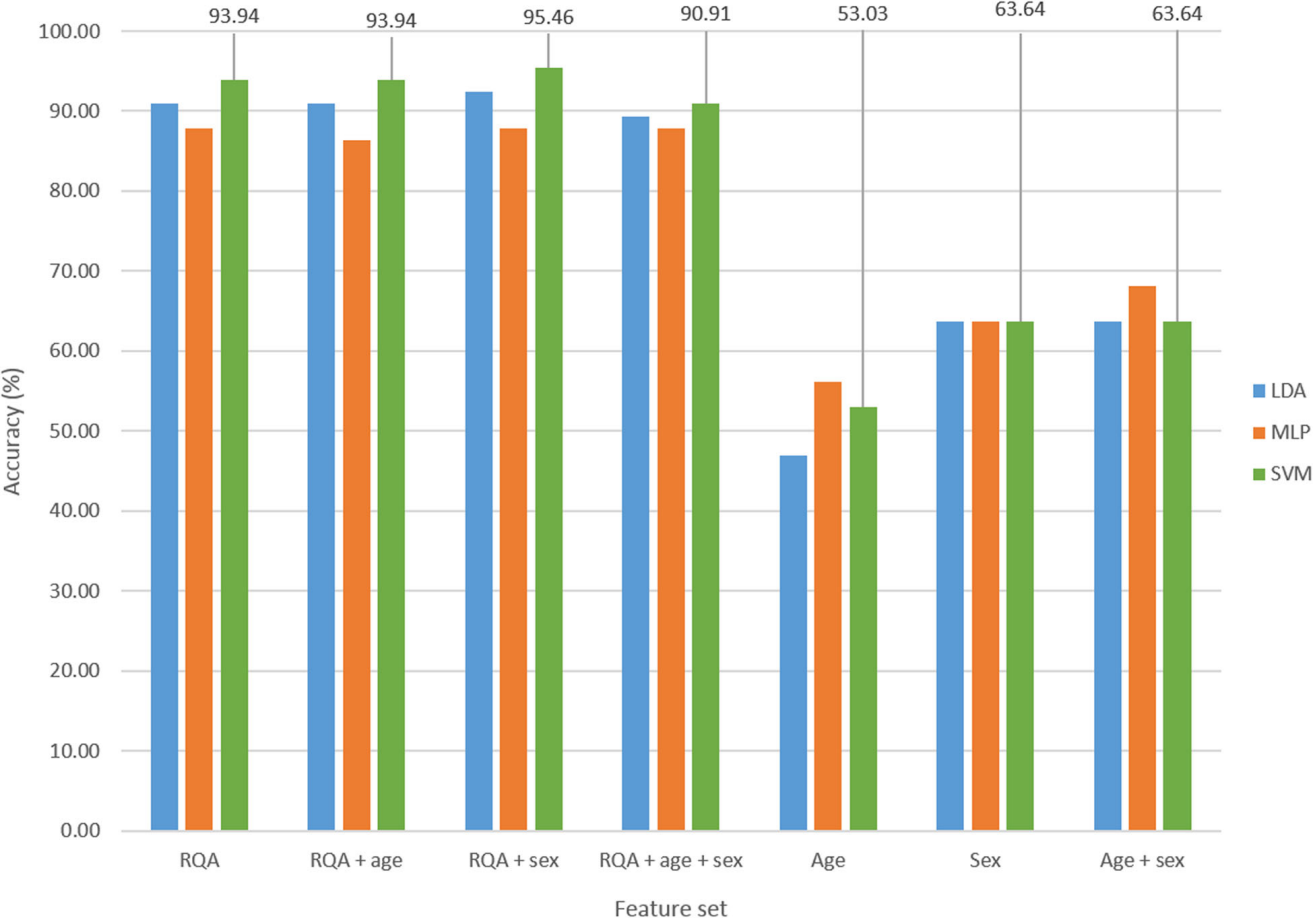
Classification accuracy of different feature sets for age-matched sample cross-validation run 1

Classification accuracy results are depicted in Fig. 5. The SVM classification results for the first cross validation run showed 93.94% accuracy with the ‘RQA’ feature set, 90.91% with ‘RQA + age + sex’, 53.03% with ‘age’, 63.64% with ‘sex’ and 63.64% with ‘age + sex’. The use of an age-matched sample with rounded down ages therefore significantly minimised the previously achieved spurious results highlighted above.

**Fig. 6:**
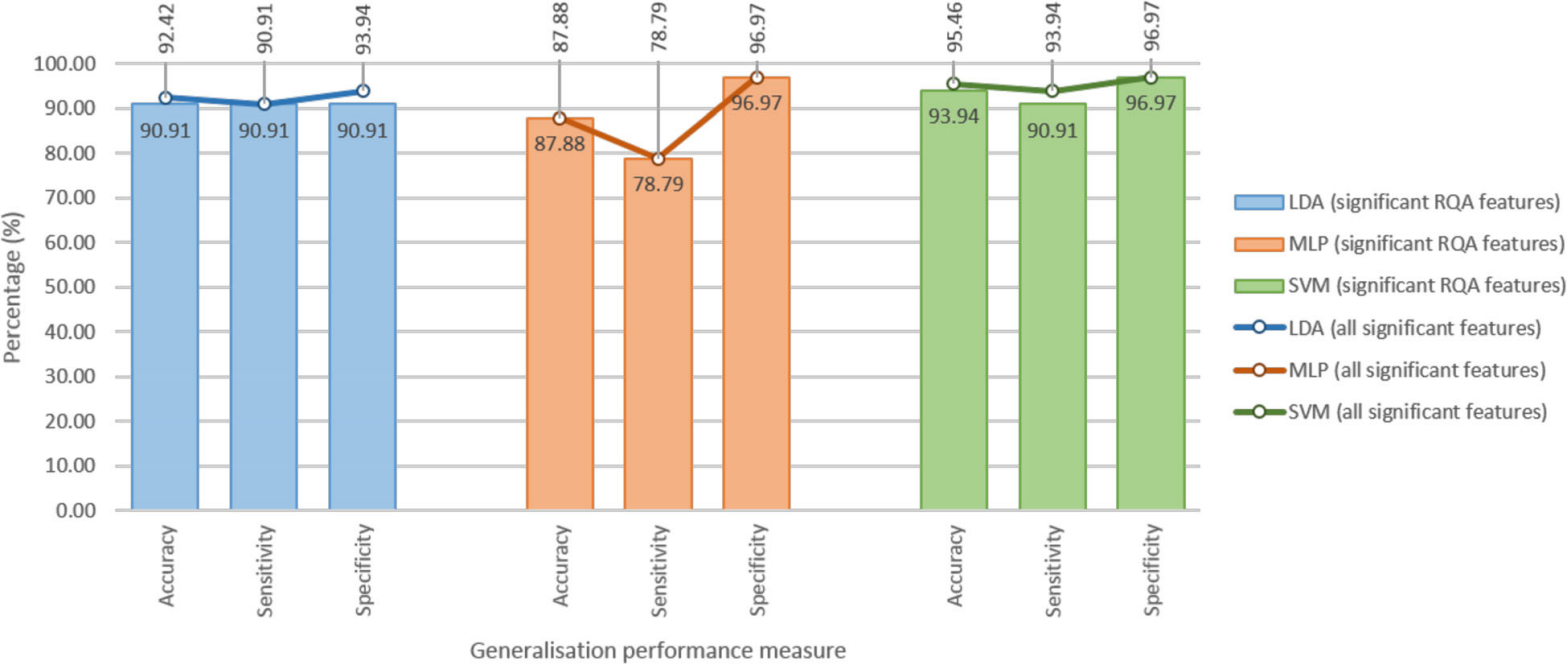
Generalisation performance for age-matched sample cross-validation run 1

Figure 6 provides an illustration of the generalisation performance (accuracy, sensitivity and specificity) achieved with each of the classifiers on feature set 1 (significant RQA features) and feature set 2 (all significant features, RQA and demographic). Both sensitivity and specificity measures are important – an ideal diagnostic test would be 100% sensitive and 100% specific, whereas a good screening tool may be more sensitive than specific. The SVM showed the best generalisation performance, with acceptable sensitivity and specificity. Feature set 1 comprised 6 RQA features, and feature set 2 comprised 6 RQA features plus sex. With addition of sex, the SVM accuracy increased from 93.94% to 95.46%, sensitivity increased from 90.91% to 93.94%, and specificity remained constant at 96.97%.

**Fig. 7:**
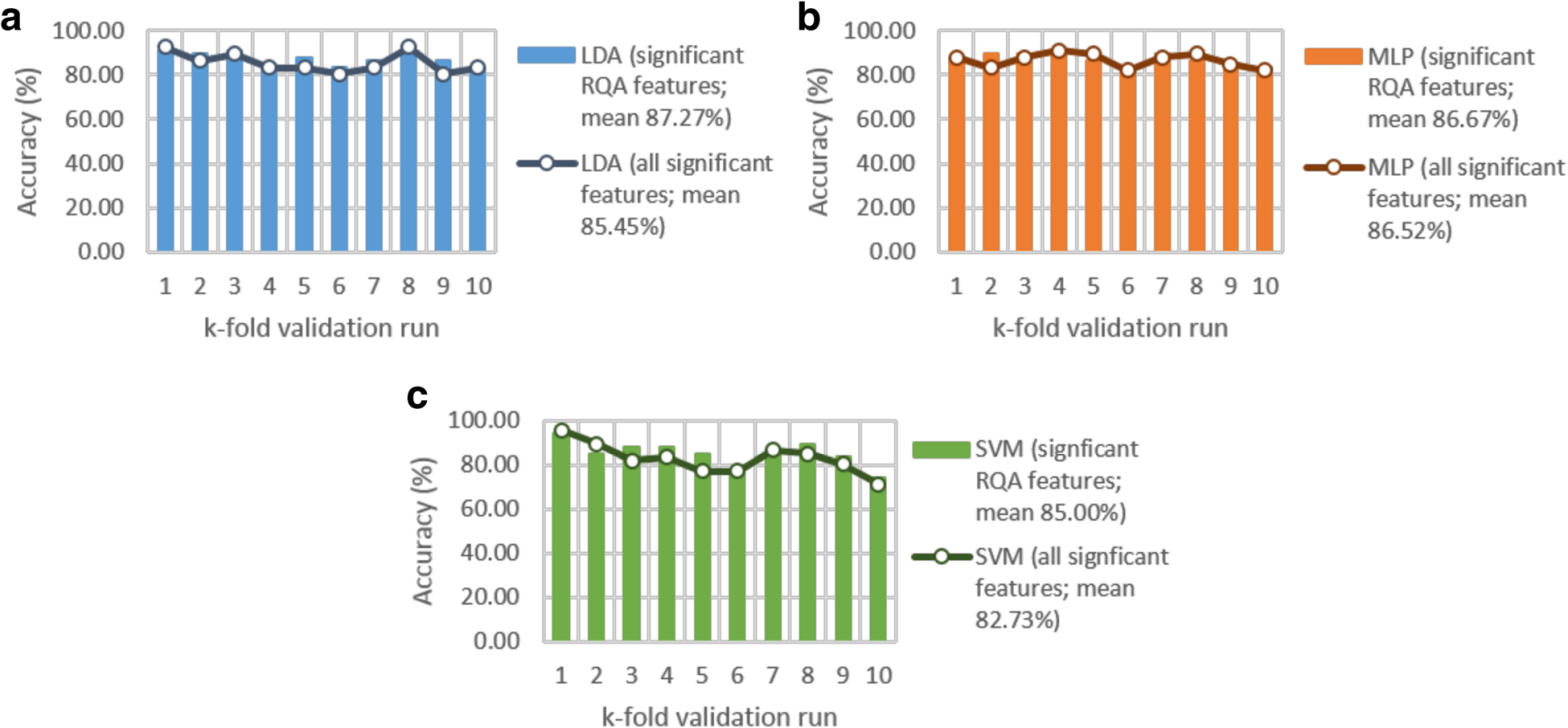
Cross-validation performance for the (**a**) linear discriminant analysis (LDA), (**b**) multilayer layer perceptron (MLP) and (**c**) support vector machine (SVM) classifiers

The 10-fold cross-validation results (Fig. 7) revealed that classification of feature set 1 (all statistically significant RQA features) outperformed that of feature set 2 (all statistically significant RQA and demographic features). A mean classification accuracy of 87.27% was achieved with LDA, 86.67% with the MLP, and 85% with the SVM, with feature set 1.

**Fig. 8:**
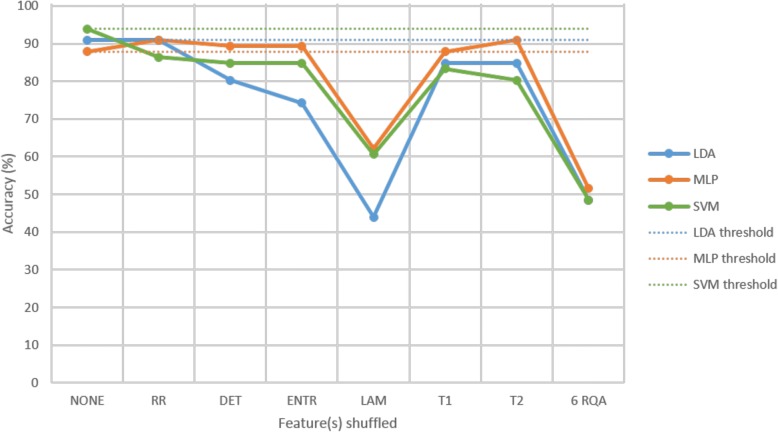
Feature shuffle analysis of feature set 1 for age-matched sample cross-validation run 1

The feature shuffle analysis results (Fig. 8) show that LAM was the most important feature to all three classifiers, as can be seen by the prominent drop in classification accuracy when the test feature set with shuffled labels for LAM was classified. This feature is indicative of the occurrence of laminar states, i.e. the probability that a state will not change for the next time step. Each of the six significant RQA features was important to the SVM classifier, evident from the drop in classification accuracy for each case. Upon shuffling the test labels of all six significant RQA features at once, an accuracy of approximately 50% was achieved, confirming that all features were contributing significant discriminatory information to the classifiers.

**Fig. 9:**
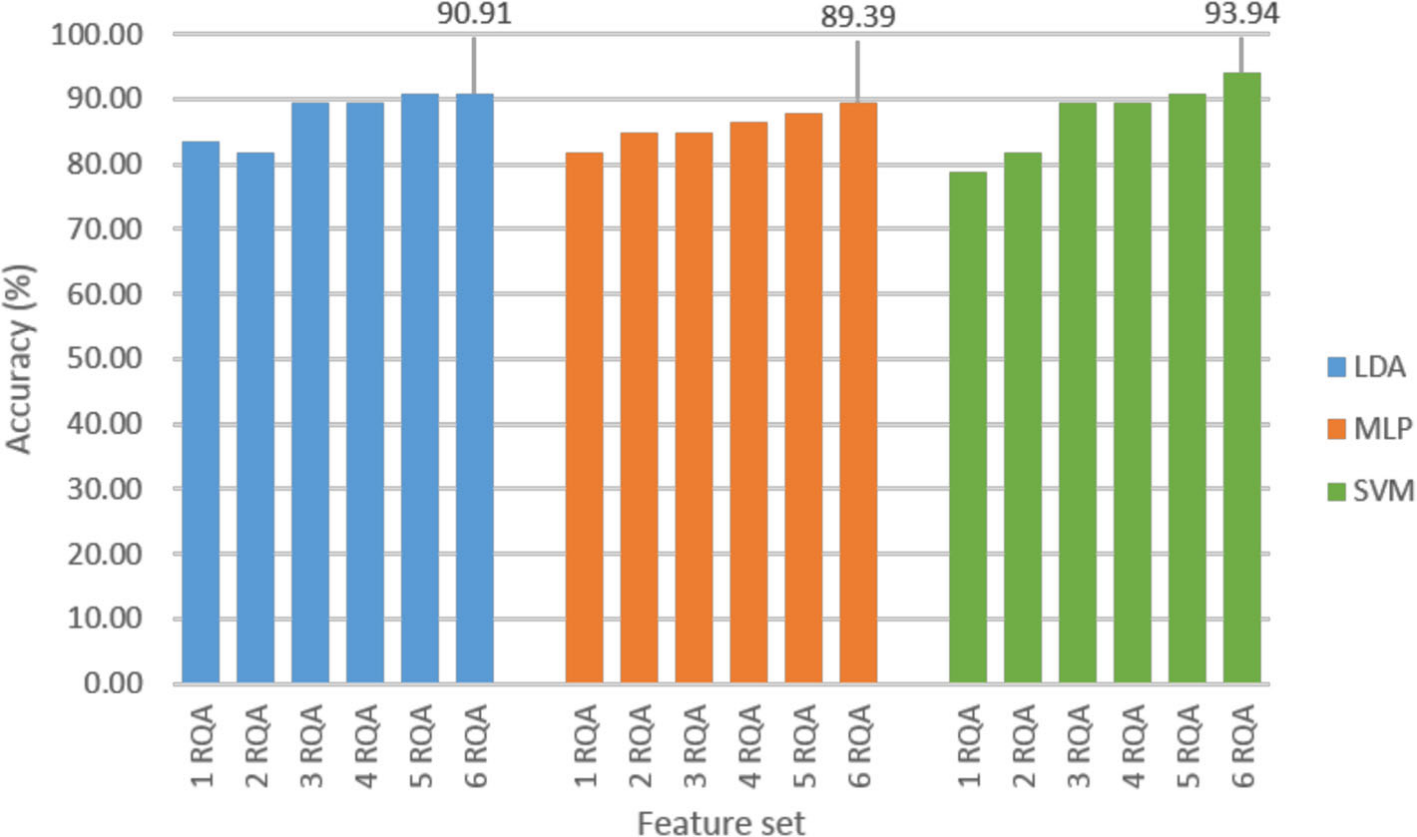
Optimal feature subset identification for age-matched sample cross-validation run 1

Classification of the individual features with the SVM showed 78.79% with LAM, 78.79% with DET, 69.70% with ENTR, 65.15% with T2, 62.12% with RR and 59.09% with T1. Identification of the optimal feature subset revealed that the highest classification accuracy was achieved when including all six of the statistically significant RQA features (Fig. 9). In this figure ‘1 RQA’ represents the LAM feature, ‘2 RQA’ represents LAM + DET, …, and ‘6 RQA’ represents LAM + DET + ENTR + T2 + RR + T1.

**Fig. 10:**
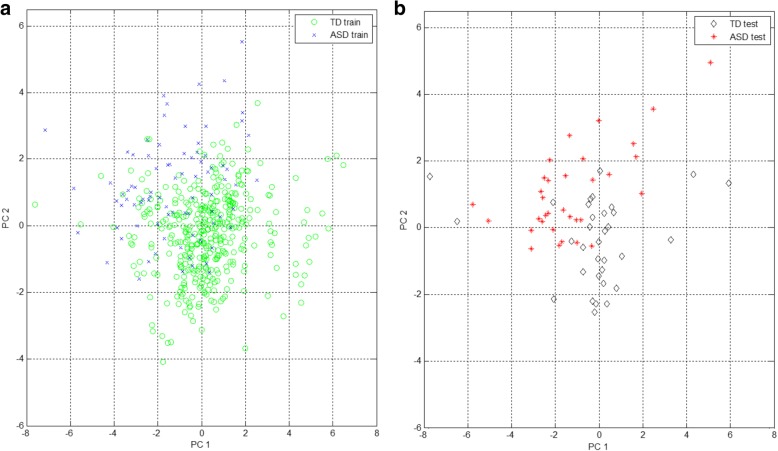
Visualisation of the feature space in a 2D principal component (PC) subspace, (**a**) training features and (**b**) test features for age-matched sample cross-validation run 1

**Fig. 11:**
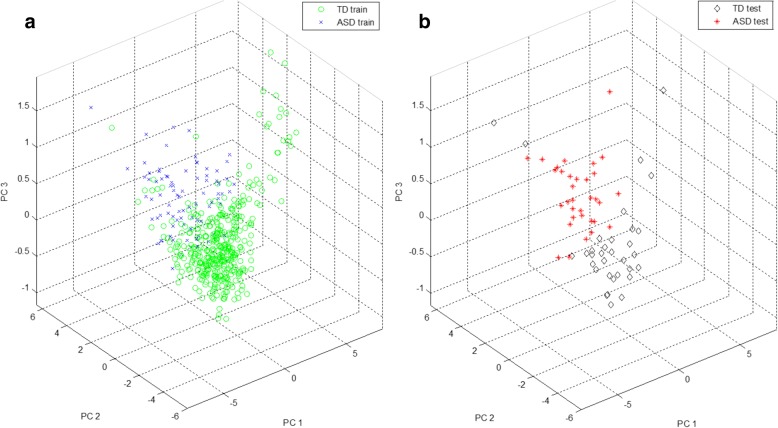
Visualisation of the feature space in a 3D principal component (PC) subspace, (**a**) training features and (**b**) test features for age-matched sample cross-validation run 1

The optimal RQA feature subset comprised six RQA features, representing a point (or state) in a six-dimensional feature space. PCA was used to enable visualisation of this multi-dimensional feature space by projecting the data to a 2D and 3D PC subspace. It is important to keep in mind that PCA is not optimised for class separation in a low-dimensional representation of the data, instead, it linearly transforms the data to a new set of orthogonal axes for which each subsequent component attempts to account for the maximum remaining variance in the data. Figures 10 and 11 provide 2D and 3D representations of the data for cross-validation run 1 in the PC subspace, accounting for approximately 94% and 99% of the variance in the data, respectively. PC directions were determined based on the training data features; test data features were then projected to this PC subspace. In the 2D representation of the feature space, it is difficult to visually distinguish the ASD and TD groups, but in the 3D representation, the separation becomes clearer. There appears to be some overlap present in samples from both groups.

**Fig. 12:**
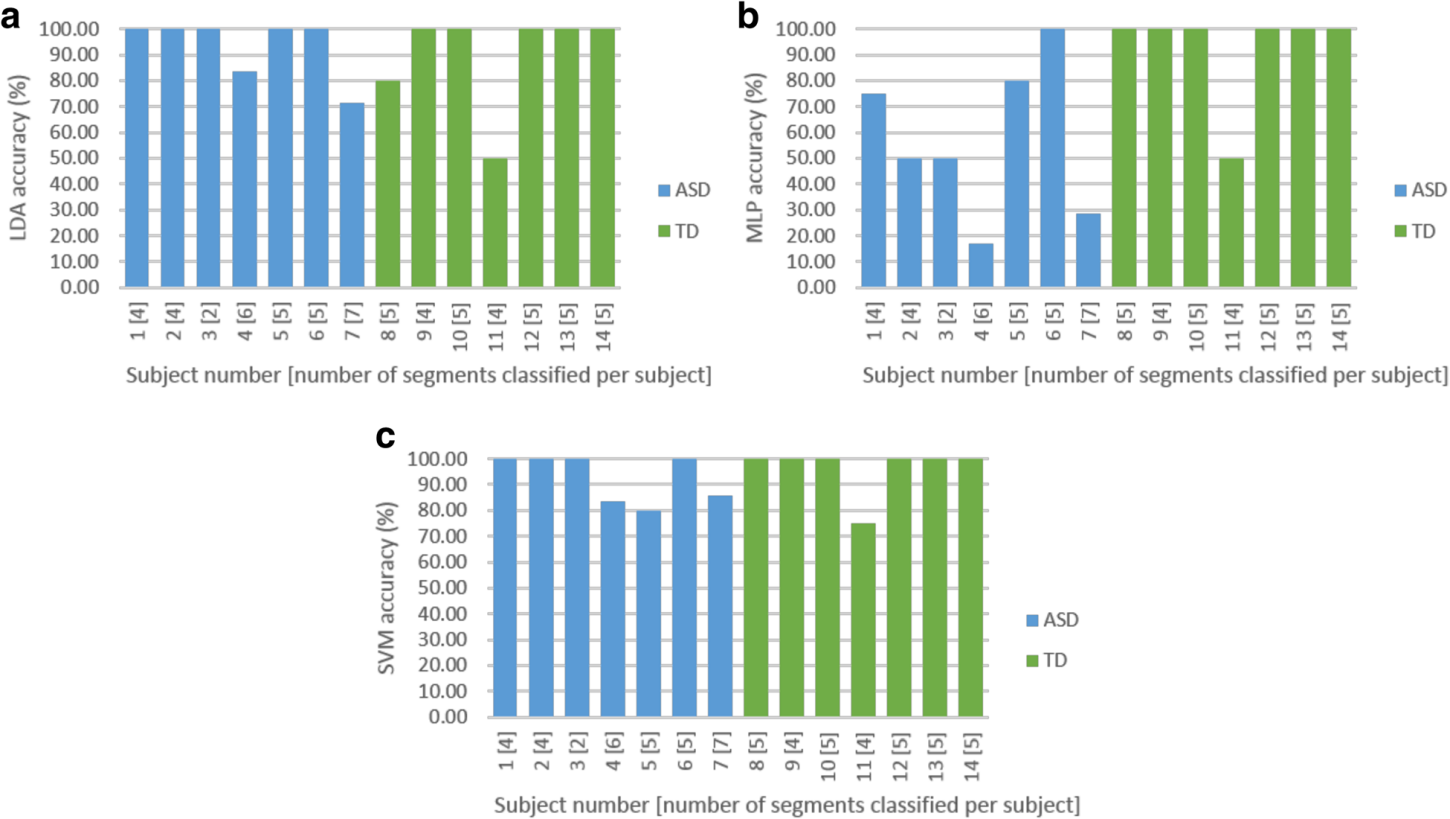
Repeatability analysis for the (**a**) linear discriminant analysis (LDA), (**b**) multilayer layer perceptron (MLP) and (**c**) support vector machine (SVM) classifiers for age-matched sample cross-validation run 1

Test-retest reliability per subject was investigated. In classification analyses, the ‘majority vote’ is often used to identify the predicted label of a test case. This was quantified with a 50% threshold. In the case where 50% or more was achieved in repeatability accuracy for all segments correctly classified per subject, the subject was considered to be correctly identified. The SVM classifier identified 4 out of 7 ASD and 6 out of 7 TD subjects with 100% accuracy. Both LDA and SVM classifiers yielded similar overall repeatability performance with the correct identification of all segments from 10 out of 14 subjects (Fig. 12).

### Age-matched sample: leave-one-subject-out

**Fig. 13:**
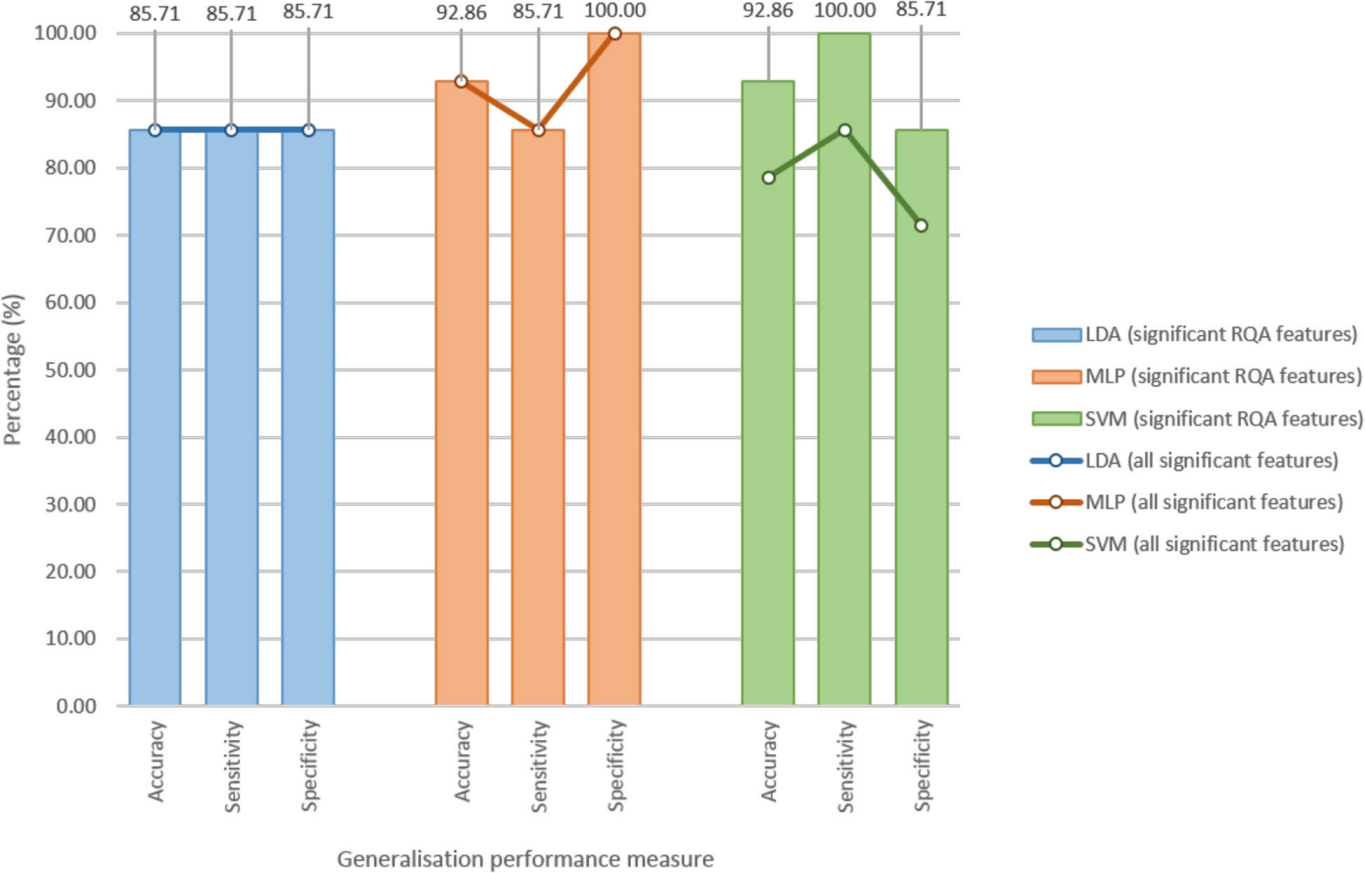
Generalisation performance for age-matched sample leave-one-subject-out analysis

**Fig. 14:**
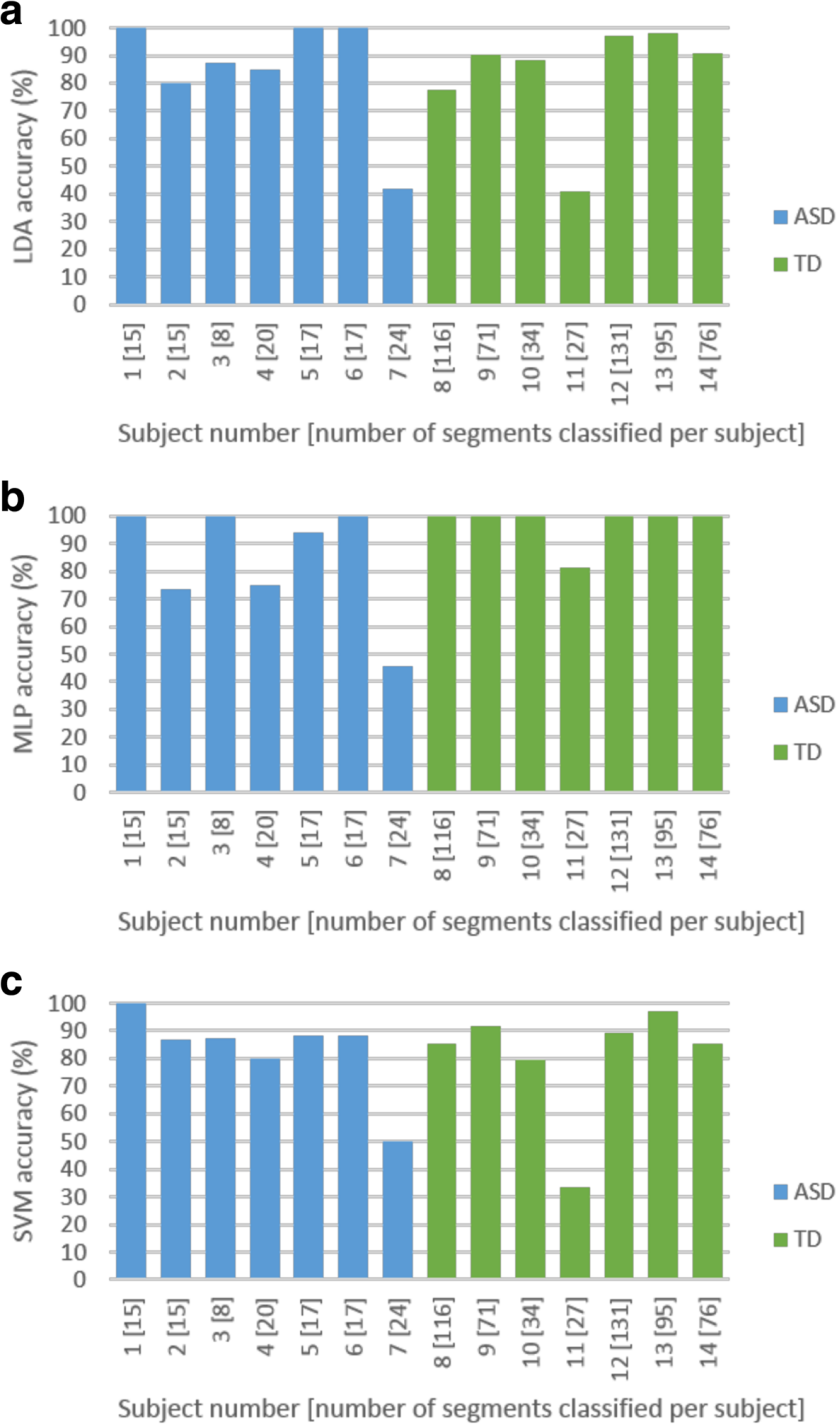
Repeatability analysis for the (**a**) linear discriminant analysis (LDA), (**b**) multilayer layer perceptron (MLP) and (**c**) support vector machine (SVM) classifiers for age-matched sample leave-one-subject-out analysis

The optimal parameter and feature set identified using the first cross validation run was used. A neighbourhood size of 2.9 was equivalent to 6.7% of the average maximum phase space size across all 14 leave-one-subject-out runs. The MLP and SVM classifiers achieved 92.86% accuracy (13/14 subjects correctly identified) with full feature set 1, with the SVM classifier being more sensitive and the MLP classifier more specific (Fig. 13). The SVM classifier achieved 100% sensitivity (7/7 ASD subjects correctly identified) and 85.71% specificity (6/7 TD subjects correctly identified), whilst the MLP classifier achieved 85.71% sensitivity (6/7 ASD subjects correctly identified) and 100% specificity (7/7 TD subjects correctly identified). Sensitivity and specificity measures are equally important, but considering the implementation of a screening test, provided two classifier options, the classifier with the higher sensitivity would be chosen, the argument being that it is safer to refer a ‘TD’ person for a second step to confirm a diagnosis, rather than to send away an ‘ASD’ person who will then fail to receive any intervention because of being considered ‘TD’. The SVM classifier would thus be most appropriate for this application. Figure 14 shows that test-retest reliability remained a challenge. The larger number of TD segments could have biased the classifiers towards the TD class, but the results suggest that this effect was negligible, as the misclassification of the ASD subjects was insignificant.

## Discussion

Early identification and diagnosis of ASD is a challenge worldwide, but particularly so in low-resource environments. There is a great demand for screening tools to identify infants and children at risk of ASD that do not require highly trained professionals and that are ‘language free and culturally fair’ [3, 9–11, 19]. Here, we evaluated RQA of rsEEG as a potential novel biomarker for ASD risk given its ability to perform multivariate analysis of short, nonlinear and nonstationary segments of rsEEG. Given the existing challenges of potential confounders to biomarkers for ASD and related neurodevelopmental disorders, we deliberately set out to scrutinise the biomarker against three key potential confounders of age, sex and intellectual ability.

The analysis progressed stepwise from a full sample, comprising 4802 5-s segments of rsEEG data from 62 subjects, stepwise, to an age-matched sample, comprising 666 segments from 14 subjects, in order to examine and eliminate possible confounding effects such as sample bias, mismatched number of test segments and unrounded ages. In the final age-matched sample, the RQA biomarker showed good accuracy, sensitivity and specificity in distinguishing ASD and TD subjects. The SVM classifier showed robust performance on the significant RQA feature set, with 92.86% accuracy, 100% sensitivity and 85.71% specificity using a leave-one-subject-out approach, which simulates the clinical scenario of diagnosing an unseen subject. Age, sex and intellectual ability were confirmed as confounders. Consistent repeatability, i.e. the correct identification of all segments per subject, was found to be a challenge.

The results presented herein highlight various obstacles to be considered when performing classification analyses. In assessing classification performance, it is important to report not only accuracy, but also sensitivity, specificity, sample size and sample composition (the proportion of samples within each group) within the training and test data sets. As illustrated here, in the full sample, 93.9% of the test data belonged to TD subjects. With classification of this sample, a classifier would have been able to correctly predict group membership 93.9% of the time by merely guessing that all segments belonged to the TD group. Consideration of the accuracy alone would yield apparent good performance, but sensitivity (with respect to the correction identification of ASD subjects) would have been zero. Statistical significance testing of the identified features, showing mean ± standard deviation values and *p* values, can provide further support to confirm classification results. A full sensitivity analysis on parameter selection will provide an additional measure of robustness.

Results showed that investigation of age as a covariate, using exact age values (e.g. 5.25) in a small sample, led to the finding that age was a good predictor in distinguishing ASD and TD subjects. In a population-based random sample, age should not be a predictor of ASD or TD. By rounding down the ages, this confounding effect was eliminated. It is vital that biomarker performance is assessed in well-matched samples where possible confounding factors, such as age, sex and intellectual disability, are carefully controlled for.

Keeping in mind the ultimate goal of biomarker translation into clinical practice, an important next step would be to establish a reliable repeatability accuracy threshold when evaluating several segments per subject to make a prediction of ‘at risk for ASD’ or not. Furthermore, it would be important to investigate the possible causes of poor test-retest reliability, such as artefact contamination, and to evaluate whether or not the use of different EEG data acquisition systems has any significant effect on the features extracted. It is beyond the scope of this study to speculate how the RQA features are associated with underlying neurophysiological phenomena.

Recently, Bosl et al. [35, 36] applied RQA and MME methodologies in a study of ASD and absence epilepsy and for early identification of children at risk of ASD as an elaboration of their earlier work [22]. They proposed that RQA methods may indeed be useful as biomarkers for ASD, thus replicating our earlier proof-of-principle findings [23]. However, they did not elaborate on technical and other potential confounders, such as sex or intellectual ability, in their work. In this study, we explored the utility of RQA to differentiate between ASD and typical development when neither group had any seizure disorders. The novelty of this current study lies in the replication and extension of the proof-of-principle study [23] in a larger sample, and to investigate the robustness of the RQA biomarker in the context of a number of clinical variables that may act as covariates or confounders [3]. Further unique contributions of this manuscript are (1) that a leave-one-subject-out classification approach was followed, simulating the clinical scenario of ‘diagnosing’ an unseen subject, and (2) a test-retest reliability analysis was performed, determining the accuracy of correctly classifying several segments per subject. Bosl et al. [35] did not implement a leave-one-subject-out classification approach (they implemented 10-fold cross validation), and they also did not investigate test-retest reliability (they analysed only a single segment per subject). Bosl et al. [35] analysed a single 30-s segment from 91 subjects, amounting to a total of 91 segments. We analysed a total of 666 5-s segments from 14 subjects.

A head-to-head comparison of the three proposed biomarker methods, MME, CA and RQA, on a common dataset may generate empirical evidence for a direct comparison to be made on biomarker performance. In order to replicate the exact methodologies reported in the literature, it is important that clear guidelines on technique selection, parameter choice, and method implementation be published alongside the results in a paper. It is also necessary to include details of the inclusion, exclusion and matching criteria implemented, the demographic distribution within the sample, and statistical testing for possible confounding factors.

## Conclusions

RQA may be an accurate, sensitive and specific biomarker to identify children at risk of ASD. In particular, the leave-one-subject-out method that mirrors a clinical decision (classification) supports this. A key aspect for a biomarker is that it should be able to classify individual cases, not groups of cases, which is the case for so many biomarkers currently proposed for ASD. However, given the heterogeneous nature of ASD, it will be important for future studies to show not only differentiation between ASD and TD, but also differentiation of ASD from other neurodevelopmental disorders, and potentially syndromal from non-syndromal ASD.

It is clear that biomarker development is complex since various methodological, demographic, clinical and technical factors need to be taken into consideration. The results from this study highlight the importance of considering age, sex and intellectual ability as possible confounders or covariates in biomarker studies. The RQA biomarker may be a robust and reliable ‘language free, culturally fair’ technology-based solution for global screening in ASD; however, a number of challenges remain to be explored. It will be important to validate this biomarker in a well-matched larger sample population of infants and children, preferably from a low-resource setting. It will also be important that the biomarker can be computed rapidly, rather than through a computationally intensive approach.

Even if we were to identify a rsEEG screening tool for ASD, many further challenges will be faced with the implementation of such a tool in low-resource settings. Low cost, battery-powered and wireless EEG recording devices and equipment with offline analysis and storage capacity, as well as easily available ‘read-outs’ will be required. Just as important, even if the above implementation challenges are resolved, infants/children identified to be at-risk will then need to be directed to appropriate clinical diagnostic and intervention services, which could be difficult to access in low- and middle-income countries and other low-resource environments.

## Abbreviations

ADOS: Autism diagnostic observation schedule
ASD: Autism spectrum disorder
CA: Coherence analysis
DET: Determinism
DSM-IV-TR: Diagnostic and statistical manual of mental disorders
EEG: Electroencephalography
ENTR: Entropy
HRA: High risk for autism
LAM: Laminarity
LDA: Linear discriminant analysis
M-CHAT: Modified checklist for autism in toddlers
MLP: Multilayer layer perceptron
MME: Modified multiscale entropy
PC: Principal component
PCA: Principal component analysis
PVR: Percentage variance to retain
RP: Recurrence plot
RQA: Recurrence quantification analysis
RR: Recurrence rate
rsEEG: Resting state electroencephalography
SVM: Support vector machine
T1: Recurrence time of the first Poincaré recurrence
T2: Recurrence time of the second Poincaré recurrence
TD: Typically developing
